# Emerging Molecular Connections between NM23 Proteins, Telomeres and Telomere-Associated Factors: Implications in Cancer Metastasis and Ageing

**DOI:** 10.3390/ijms22073457

**Published:** 2021-03-27

**Authors:** Shalu Sharma, Antara Sengupta, Shantanu Chowdhury

**Affiliations:** 1 Integrative and Functional Biology Unit, CSIR-Institute of Genomics and Integrative Biology, Mathura Road, New Delhi 110025, India; shalu.sharma31890@gmail.com (S.S.); antara.sengupta@igib.in (A.S.); 2 Academy of Scientific and Innovative Research (AcSIR), Ghaziabad 201002, India; 3 GNR Knowledge Centre for Genome and Informatics, CSIR-Institute of Genomics and Integrative Biology, Mathura Road, New Delhi 110025, India

**Keywords:** NDPK proteins, telomere, telomere length, DNA binding, protein kinase, G-quadruplex, ageing, mitochondria, cytoskeleton

## Abstract

The metastasis suppressor function of NM23 proteins is widely understood. Multiple enzymatic activities of NM23 proteins have also been identified. However, relatively less known interesting aspects are being revealed from recent developments that corroborate the telomeric interactions of NM23 proteins. Telomeres are known to regulate essential physiological events such as metastasis, ageing, and cellular differentiation via inter-connected signalling pathways. Here, we review the literature on the association of NM23 proteins with telomeres or telomere-related factors, and discuss the potential implications of emerging telomeric functions of NM23 proteins. Further understanding of these aspects might be instrumental in better understanding the metastasis suppressor functions of NM23 proteins.

## 1. Introduction

The Non-metastasis 23/Nucleoside diphosphate kinase NM23/NDPK protein family contributes to several cellular pathways owing to their interaction with multiple effector proteins and several known enzymatic functions of the NM23 proteins. This multifunctional family of proteins (10 in humans) is broadly divided into two groups based on their identity (Group I: NM23H1-H4, 58–88%; Group II: more divergent members, 22–44%). NM23-H1 and H2, the cytoplasmic/nuclear localizing proteins, and NM23-H4, reported mostly as mitochondrial, are amongst the most studied members of the family of proteins. These proteins have previously been extensively reviewed [1,2]. The combination of nucleoside diphosphatase kinase activity, histidine protein kinase activity, DNA binding, cell motility suppression, and transcription regulatory functions has motivated the continuous exploration of NM23/NDPK proteins and their role in cell physiology [3]. In mammalian systems, this protein-family has been associated with metastasis suppression in cancers, and they are therefore commonly known as “non-metastatic” proteins [4]. Despite a large body of work, molecular mechanisms involved in the metastasis suppressor function of the NM23 family of proteins continue to be of interest. 

One of the lesser studied yet relatively direct links with cancer initiation, progression and metastasis comes from the association of NM23 proteins with chromosome ends or telomeres, and telomere binding proteins (telosome/shelterin complex) [5]. Evidence showing the association of NM23 proteins with telomeres and telosome suggests involvement of NM23 proteins in telomere maintenance. Additionally, evidence suggests the interplay of telomeres, NM23 proteins and mitochondrial dysfunction in ageing. With these in mind, here we discuss the emerging literature on direct and/or indirect functions of NM23 proteins related to telomeres and telomere-associated proteins, with particular emphasis on the role of these mechanisms in cancer and ageing.

## 2. Direct Interaction of NM23 Proteins with Telomeres and Telomere-Binding Proteins

### 2.1. NM23 Proteins Bind with the Telomeric DNA Sequence In Vitro

The first evidence of NM23 proteins interacting with the telomeric sequence came from Nosaka et al., 1998 [6]. Thirty-six -mer single and double-stranded oligonucleotides comprising telomeric repeats (TTAGGG)_6_ were incubated with Glutathione S-transferase (GST)-tagged recombinant NM23-H1 or H2. Only NM23-H2 had binding affinity for the telomeric sequence, but not NM23-H1. NM23-H2 was found to bind with the single-stranded, but not the double-stranded telomeric sequence in vitro. The authors further tested the specificity of the binding of NM23-H2 to the telomeric sequence and found that NM23-H2 associated with both (TTAGAG)_6_ and (TTGGGG)_6_, but not the (ATAGGG)_6_ sequence. 

The NM23-H2 homologue in zebrafish *Danio rerio* known as “NM23-B”, discovered based on 87.3% sequence similarity, was also found to interact with the telomeric DNA sequence (TTAGGG)_6_ [7]. Additionally, a close homologue of human NM23-H5 (NME5) recently found in the red alga *Chondrus crispus*—and therefore named NME5-likeCc—was also found to interact with telomeric DNA (TTAGGG)_6_ in vitro [8].

### 2.2. NM23-H2 Interaction with Telomere-Like G-Quadruplex Motif at the c-MYC Promoter

In 1993, Postel et al. identified the human *c-MYC* purine-binding factor (PuF)—named based on the characteristic G-rich DNA binding by the transcription factor—to be NM23-H2 [9]. Later work showed that NM23-H2 associated with the G-quadruplex DNA secondary structure formed by the G-rich DNA sequence present within the nuclease hypersensitive element (NHE) of the *c-MYC* promoter [10]. G-quadruplexes were initially noted to be formed by the interspersed GGG repeat-containing sequence found at the telomeres [11]. Similar interspersed GGG repeats identified at the *c-MYC* promoter NHE that formed telomere-like G-quadruplex, along with NM23-H2 association with telomeric DNA (discussed above), supported NM23-H2-G-quadruplex interactions. Disruption of the G-quadruplex by specific base substitutions, or in the presence of intracellular G-quadruplex binding molecules, resulted in altered binding of NM23-H2 to the *c-MYC* promoter. In addition, in vitro studies using recombinant NM23-H2 showed NM23-H2-G-quadruplex binding [10]. Recent work further shows NM23-H2 association with the G-quadruplexes at the *hTERT* promoter to be important for the epigenetic regulation of *hTERT* (*vide infra*) [12]. Interestingly, NM23-H2 was also shown to bind the i-motif formed from single-stranded C-rich DNA at the *c-MYC* promoter NHE [13].

### 2.3. NM23-H2 Interacts with Telomeres Inside Cells

**Figure 1:**
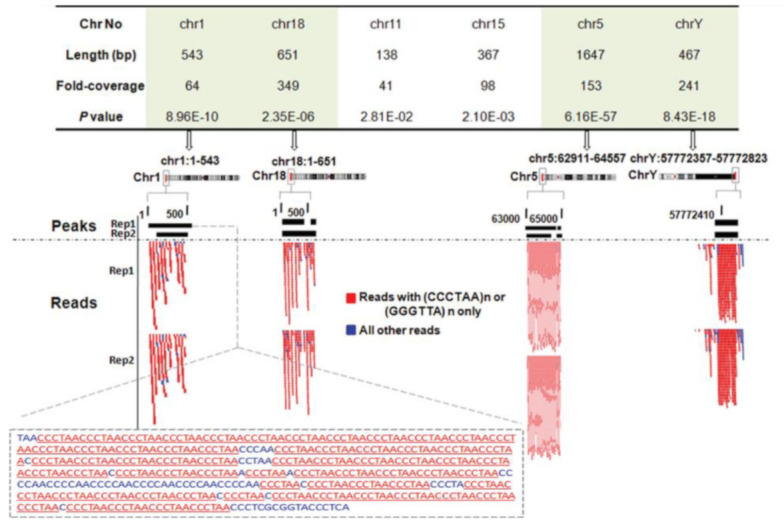
NM23-H2 ChIP-seq reads are enriched for TTAGGG or CCCTAA repeats from the telomeres. Reproduced with permission from Kar et al., 2012 NAR [5].

While the findings discussed above demonstrated the interaction of NM23-H2 with telomeric DNA in vitro, evidence supporting NM23-H2 binding to telomeres in vivo was shown in 2012. In both A549 lung carcinoma and HT1080 fibrosarcoma cell lines, NM23-H2 chromatin immunoprecipitation followed by sequencing (ChIP-seq) was performed following expression MYC-tagged-NM23-H2. On analysis of the short sequence reads, for every million ChIP-seq about 6000–9000 reads were from the telomeric region comprising three or four telomeric repeats (TTAGGG/CCCTAA) (Figure 1). Here, it is important to note that because of ChIP with the anti-MYC antibody (against MYC-tagged NM23-H2), chances of cross-reactivity with NM23-H1 were unlikely. Further, ChIP experiments for both NM23-H1 and H2 showed that while NM23-H2 had occupancy on telomeric repeats, this was not so for NM23-H1 [5]. 

### 2.4. Interaction of the Telomere Binding Factor TRF1 and TRF2 with NM23-H2

The shelterin is an essential multi-protein complex that prevents the telomere ends from being detected as DNA double-stranded breaks, thereby preventing the activation of DNA damage signalling [14]. The shelterin also mediates telomere maintenance by assisting the recruitment of *telomerase* (the specialized reverse transcriptase holo-enzyme (TERT) that synthesizes telomeres) to the telomeres [15]. Telomere repeat binding factors 1 and 2 (TRF1 and TRF2) are DNA binding proteins essential for the assembly of the shelterin [16]. 

The direct association of NM23-H1 and H2 with TRF2 was tested. Here, the authors found co-immunoprecipitation (co-IP) of TRF2 with NM23-H2 from nuclear extracts of HT1080 fibrosarcoma and A549 lung carcinoma cell lines. This was further validated using reverse-IP using pull-down with anti-TRF2 antibody, where interaction with NM23-H2 was observed. Since both NM23-H2 and TRF2 were known to interact with DNA, it was further tested if NM23-H2-TRF2 interaction was dependent on their association with DNA. NM23-H2-TRF2 interaction was found to be independent of DNA or RNA association [5]. Consistent with previous reports, no interaction was noted between NM23-H1 and TRF2.

Earlier work using the yeast two-hybrid method showed the interaction of a truncated TRF1 protein (14-285aa) with NM23-H2; the interaction of TRF1 with NM23-H1, however, was not significant. Further, the authors used recombinant GST-tagged NM23-H1 and H2 and translated full-length TRF1 with S^35^ labelled methionine in vitro to confirm a strong and direct association of NM23-H2 with TRF1. The interaction of NM23-H1 with TRF1 was again found to be insignificant [6].

### 2.5. Human Telomerase Physically Associates with NM23-H2

The ribonucleoprotein reverse transcriptase telomerase binds to the 3’ overhangs of the telomeres and processes telomere elongation [17]. Telomerase hyper-activation is found in >90% of cancers and is known to support telomere maintenance in fast-dividing tumour cells [17,18]. Like NM23-H1 and H2, telomerase has also been associated with metastasis [19,20,21,22,23,24,25,26]. Based on findings supporting the presence of NM23-H2 at telomeres, the interaction of NM23-H2 with human telomerase (hTERT) was tested using co-IP experiments. Based on results from these experiments using nuclear extracts of HT1080 fibrosarcoma and A549 lung carcinoma cells, authors concluded that NM23-H2 associates with hTERT inside cells [5]. 

Further, it was observed that NM23-H2 over-expression suppressed hTERT enzymatic activity in HT1080 fibrosarcoma cells. This was independent of the nuclease activity of NM23-H2, suggesting that NM23-H2 did not suppress telomerase activity by cleaving the template DNA necessary for telomere elongation by hTERT [5]. As expected, the prolonged over-expression of NM23-H2 resulted in telomere shortening in MDA-MB-231 breast cancer cells, further supporting the role of NM23-H3 in the suppression of telomerase activity [27]. 

While these results supported an inverse relationship between NM23-H2 and telomerase, notably, NM23-H2 and telomerase activity when tested in hepatocellular carcinoma (HCC) patient samples showed that NM23-H2 levels positively correlated with telomerase activity [28]. Here, it is important to note that NM23-H2 is mainly localized in the cytoplasm, instead of the nuclei, in hepatocellular carcinoma cells [29]. 

Other than TRF2 and hTERT, the nuclear interactome of NM23-H2 (from mass spectrometry data) showed that telomere-associated proteins flap-endonuclease-1 (FEN1) and Lamin A/C/B1 interacted with NM23-H2 [12,30,31,32]. Additionally, NM23-H1 levels were reported earlier to directly co-relate with *FEN1* mRNA in melanoma cell lines [33,34]. Further studies would be required to understand the functional implications of these newly detected interactions. 

### 2.6. NM23-H2-Mediated Epigenetic Regulation of Human Telomerase Transcription

In addition to physical interaction with telomerase, direct transcriptional control of the *hTERT* by NM23-H2 was reported. From NM23-H2 ChIP in HT1080 fibrosarcoma and HCT116 colorectal cancer cell lines, NM23-H2 binding at the *hTERT* promoter was found within +40 to -250 bp upstream of the *hTERT* transcription start site [12]. NM23-H2 silencing induced *hTERT* gene expression in HT1080 and HCT116 cancer cells, and MRC5 normal primary lung fibroblast cells. NM23-H2 mediated transcriptional repression of *hTERT* was dependent on its DNA binding activity: the N69H and R34A DNA binding mutants of NM23-H2 could not repress *hTERT* expression. The authors also observed that NM23-H2 binding was dependent on the *hTERT* promoter G-quadruplex. In the case of base substitutions that disrupted the *hTERT* promoter G-quadruplex, NM23-H2 binding was lost from the *hTERT* core-promoter. This also resulted in the loss of the NM23-H2-mediated suppression of *hTERT* [12].

**Figure 2:**
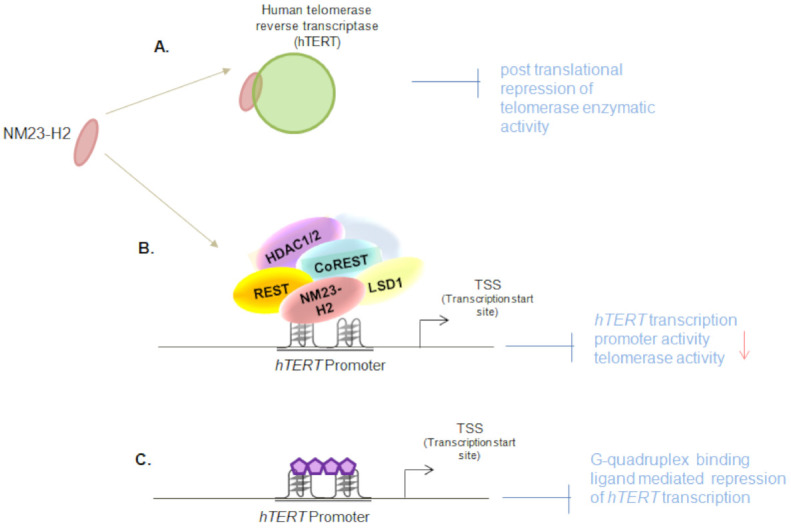
Dual regulation of human telomerase reverse transcriptase (hTERT) by NM23-H2: (**A**) NM23-H2 associates with the hTERT protein and represses the telomerase enzymatic activity [5]; (**B**) NM23-H2 directly binds the *hTERT* promoter and represses *hTERT* transcriptionally by recruiting REST/co-REST/LSD1/HDAC1/2 repressor complex on the *hTERT* promoter in a G-quadruplex dependent manner; (**C**) G-quadruplex binding ligands alter *hTERT* promoter epigenetics similar to G-quadruplex binding protein, NM23-H2 [12].

More detailed mechanistic experiments showed that NM23-H2 interacts with the REST/co-REST/LSD1/HDAC1/2 repressor complex, and this retained the non-permissive epigenetic state of the *hTERT* promoter. While lysine-specific demethylase 1 (LSD1) binding on the *hTERT* promoter was reported previously, this study demonstrated that LSD1 was recruited onto the *hTERT* core-promoter by NM23-H2. Based on the findings from the NM23-H2 nuclear interactome (mass-spectrometric analysis), and because LSD1 was known to be a part of the REST/co-REST/LSD1/HDAC1/2 repressor complex, the interaction of NM23-H2 with the remaining members of the multiprotein repressor complex was tested. Co-Immunoprecipitation experiments with anti NM23-H2 antibody showed enrichment for REST, LSD-1, HDAC1 and HDAC2. In the presence of NM23-H2, the repressor complex was shown to remain bound to the *hTERT* promoter. To further test the effect of NM23-H2 on the repressor complex, NM23-H2 was silenced. This resulted in a gain of H3K4me1, H3K4me2, and H3K9ac activation marks onto the *hTERT* promoter, as expected [12]. Taken together, these show that NM23-H2 suppresses telomerase transcriptionally as well as post-translationally (Figure 2). 

Further, the authors treated cancer cells with G-quadruplex stabilizing ligands and demonstrated their repressive effect on *hTERT* expression. The effect was further confirmed using *hTERT* promoter activity. These data highlight the potential of G-quadruplex stabilizing ligands as anti-cancer therapeutic agents that could function by telomerase suppression. In this context, it would be interesting to test the effect of G-quadruplex stabilizing ligands on cancers with *hTERT* promoter mutations [35,36], more so since these mutations are single base transitions of G > A and are known to disrupt the *hTERT* promoter G-quadruplex [37,38,39].

## 3. Putative Telomere Dependent and Independent Role of the NM23NDPK Variants in Ageing and Cancer 

### 3.1. Potential Role of NM23-H4 in Regulation of Ageing 

NM23-H4 (also known as NDPK-D) has been reported for its dual functions as a mitochondrial nucleoside diphosphate kinase and a mediator of selective membrane phospholipid transfer [40]. As a mitochondrial nucleoside diphosphate kinase, it maintains the GTP pool for the dynamin-like mitochondrial GTPase Optic Atrophy 1 (OPA1) [40]. Further, NM23-H4 was shown to facilitate the externalization of the mitochondrial inner membrane phospholipid cardiolipin (CL) in response to stress, regulating mitophagy [41,42]. Lipid-mediated mitophagy has been observed to be important in regulating mitochondrial quality checks in neuroblastomas and neuronal cells, and its dysregulation is associated with age-related neurodegenerative disorders, such as Alzheimer’s disease [43,44]. It is of interest to note that while an enzymatically active NM23-H4 was required for interaction with OPA1, binding to and externalization of CL was through a kinase-inactive form of NM23-H4 [40,41]. A report also suggests the nuclear localization of NM23-H4 mediated by SIRT1, an NAD^+^ dependent deacetylase, by modulating the acetylation levels of the protein in N1E-115 mouse neuroblastoma cells [45]. However, these assays were performed by overexpressing Myc-tagged NM23-H4 and HA-tagged SIRT1, and hence require further confirmation with endogenous proteins. These indicate a possible differential temporal localization of the different NM23-H4 forms inside the cell, reflecting the status of cellular health and age. The mitochondrial NM23 protein also regulates mitochondrial DNA (mtDNA) stability by maintaining organellar deoxyribonucleotide-triphosphate (dNTP) metabolism, further regulating reactive oxygen species (ROS) generation, telomere, and chromosomal stability, which are important early events in ageing and cancer [46,47].

### 3.2. NM23-H1 Levels Alter during Ageing-Related Disorders

Reports of NDPK family proteins associated with age-related disorders suggest the significance of NDPK in the physiological process of ageing. Based on increased specific activity in brain tissue compared to others during embryonic development, NDPK proteins were deemed to be important for neuronal functions and developmental stages in the late 1990s [48]. More recent proteomic analyses (2D gel electrophoresis followed by Matrix assisted lazer desorption/ionization mass-spectrometery (MALDI-MS) of tissues from different brain regions of patients with Alzheimer’s disease (AD) and Down’s Syndrome (DS) with AD-like neural pathology presented an important observation: NM23-H1 (also known as NDPK A) expression is substantially reduced in the frontal, parietal and occipital cortices. In addition, the enzymatic activity of NM23-H1 was also found to be reduced compared to the control. However, though no correlation could be established between reduced protein levels and activity, the study reasoned that the possibility of oxidative modification of NM23-H1 gave reduced enzymatic activity. This is concordant with the increased oxidative stress symptoms of AD [49]. Together, the maintenance of NDPK levels in specific tissues might be important in ageing and ageing-related diseases. Higher nuclear levels of NM23-H1 were also found to serve as a better prognostic marker for laryngeal squamous cell carcinoma (LSCC) in elderly patients [50]. The clinical study found around 60% (31 out of 54) of surgical specimens of LSCC patients to have nuclear NM23-H1 localization and a significantly longer disease-free survival (DFS) in patients with nuclear expression above a Receiver Operating Curve (ROC) determined cut-off level of 2%. Although a mechanistic explanation for better LSCC prognosis via age-dependent nuclear NM23-H1 expression was not explored, the possibility of its role in DNA binding and ageing-associated DNA damage-repair/response was highlighted [34,50]. 

### 3.3. Cytoskeleton Modulation by NM23/NDPK Proteins in Cancer and Ageing

Cytoskeletal modulations play a critical role in cellular and organismal ageing, neural degeneration, and cancer [51,52]. Various NDPK family proteins have been extensively reported to be associated with all the three major cytoskeleton components—actin, microtubules, and intermediate filaments, and their interacting proteins [53]. These interactions were also noted to be significant in regulating cell division and migratory movements in cancer. The interaction of gelsolin (actin-depolymerizing protein) with NM23-H1 was reported to inhibit actin filament generation, and this was associated with the metastasis inhibitory function of NM23-H1 [54]. NM23-H1 also binds to dynamin (a GTPase) involved mainly in E-cadherin endocytosis that regulates migratory/invasive properties in tumours [55]. During telophase-cytokinesis, NM23-H1 localizes in the cellular cortex, specifically at the equatorial region, wherein by binding to dynamin-1 it facilitates the formation of the cleavage furrow via actin organization [56]. Failure to do so leads to the generation of aneuploid cells, chromosomal instability, and tumourigenesis, and induces senescence in p53 expressing primary cells [56]. It would be important to note here that damage to the telomeres, which tether chromosomes to the nuclear envelope in a laminA/C dependent fashion through the linker-of-nucleoskeleton-and-cytoskeleton (LINC) complex SUN1 and SUN2 during telophase, inhibits cytokinesis [57,58]. As discussed earlier, NM23-H2 associates with lamin-A, suggesting that connections among telomeres, NM23H1/H2, and the cytoskeleton might be of significance in ageing [12,59,60]. Stathmin (Op 18), a microtubule destabilizing protein important for neuronal differentiation, was reported to be regulated by NDPK [61]. Interestingly, in addition to the drop in NM23-H1 expression in AD and DS disorders, Op18 expression also decreases. Together, these make a case for the involvement of NDPK family proteins in ageing [62]. 

## 4. Role of NM23/NDPKs in T-Cell Activation—Potential Telomerase Connection

### 4.1. NM23-H2/NDPK-B Mediated T Cell Activation

T cell activation is an indispensable arm of the adaptive immune response against foreign antigens. Factors and mechanisms governing the activation and amplification of T cells have been studied extensively. T cells derive the name from “Thymus”, where they are generated and programmed specifically for a particular antigen. T cells exit from the Thymus and enter into circulation, where they recognize their specific antigen presented on the surface of antigen-presenting cells (APCs) held by the Major histo-compatibility (MHC) complex. Antigen and T cell receptor (TCR) binding both in CD4+ helper and CD8+ cytotoxic T cells triggers T cell activation [63,64,65]. Antigen interaction is known to be accompanied by calcium (Ca^2+^) influx as an essential signal for the reactivation of naïve (in-activated/resting) T cells [66].

Ca^2+^ influx into T cells occurs via Ca^2+^ release-activated channels (CARC) present in the plasma membrane. CARC channels function by activating K+ channels (calcium-activated K+ channels, also known as IK Ca^2+^, small conductance calcium-activated potassium channel KCa3.1 ((SK)4, or KCNN4)). Therefore, CARC channels mediate Ca^2+^ influx via K+ efflux. Increased cytosolic Ca^2+^ activates phosphatase calcineurin, which mediates the assembly of Nuclear factor of activated T cell (NFAT) transcriptional complexes [66]. This initiates the transcription of genes essential for T cell activation and proliferation. 

NDPK-B/NM23-H2 was shown to activate KCa3.1 by directly binding and phosphorylating histidine (358) at its carboxyl terminus. H358 phosphorylation by NDPK-B was found to be essential for KCa3.1 channel activation, Ca^2+^ influx, and the proliferation of human CD4 T cells [67]. This study provided one of the first functions of histidine phosphorylation in mammals, governing an essential biological pathway. Later, the same group developed NDPK-B/NM23-H2 null (−/−) mice. These mice were phenotypically normal at birth and had a normal life span, with normal T and B cell development. However, cytokine production and T cell activation were markedly defective in NDPK-B (−/−) mice. The authors identified that KCa3.1 channel activity was significantly compromised in both T helper 1 (Th1) and Th2 cells. Additionally, they isolated T cells from NDPK-B (−/−) mice and tested them in-vitro. T cell activation was found to be impeded due to Kca3.1 channel dysfunction [68]. Further NDPK-B silencing was shown to abrogate T cell activation [67,69]. Together, these highlight the importance of NDPK-B in KCa3.1 function, and thereby T cell activation.

### 4.2. Potential Role of NM23-H2 in Telomerase Re-Activation during T Cell Activation

The molecular mechanisms underlying calcium signalling in T cell activation have been known for a long time [66]. Telomerase regulation and changes in telomere length during T cell activation have been studied [70,71,72,73]. Like other somatic cellular lineages, immune cells also undergo telomere shortening with cell divisions and ageing. T cell activation induces cells for further proliferation. Consistent with this, telomerase was found to be re-activated with T cell activation [74,75,76]. In a study conducted with six T cell subsets from 111 human adults, both *hTERT* mRNA and telomerase activity were found to sequentially decrease from naïve (TN) to central memory (TCM) to effector memory (TEM) cells. CD4+ cells had higher telomerase in comparison to the corresponding CD8+ subsets [76]. The robustness of T cell response was also found to be directly related to the telomere length of patient-derived activated T cells. This suggests that the gain of telomerase expression could be used to therapeutically improve the T cell function in the elderly [76,77,78]. These studies underscore the importance of telomerase and telomere length regulation in T cell response. T cell activation by phorbol myristate acetate (PMA)/ionomycin was shown to activate telomerase activity as well as calcium influx [66,70,71,72,73]. In addition to the above, while not directly in T cells, the effect of cytoplasmic calcium levels on telomere length has been demonstrated [79]. 

More than one mechanism of telomerase re-activation has been implicated to be involved during T cell activation. These include cytokine-mediated (IL-7 and IL-15) upregulation and NFAT-sp1 mediated transcriptional re-activation, and while not tested in T cells, the role of c-myc in telomerase re-activation has also been discussed, along with the post-transcriptional stabilization of telomerase transcripts (reviewed in [80,81,82]. While multiple mechanisms have been implicated, none of them have been directly elucidated to control telomerase re-activation during T cell activation. Although not in T cells, NM23-H2/NDPK-B was also reported to transcriptionally as well as post-transcriptionally suppress telomerase [5,12]. 

**Figure 3:**
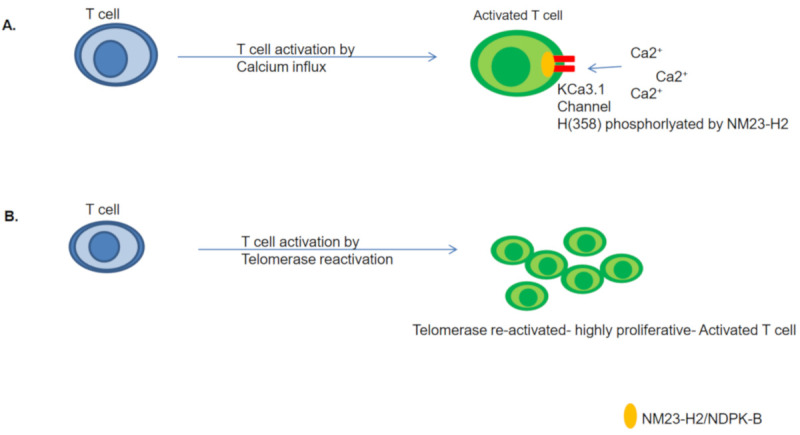
NM23-H2 could be mediating T cell activation by governing Ca^2+^ influx along with telomerase re-activation. (**A**) NM23-H2/NDPK-B phosphorylates KCa3.1 channel at Histidine 358 position, mediating T-cell activation [66], (**B**) Telomerase re-activation by depletion of NM23-H2 might induce hyper-proliferation of activated T cells [69,70,71,72].

It is possible, therefore, that the NM23-H2 mediated transcriptional regulation of *hTERT* might be of significance in the regulation of telomerase in T cells. Further, as described above, the possibility of T cell activation being dependent on Ca^2+^ influx mediated by NDPK-B/NM23-H2 kinase activity is interesting (Figure 3). Together, these implicate a dual role of NM23/NDPK proteins in T-cell activation. 

## 5. Emerging Aspects and Future Questions

The binding of NM23 proteins to telomeres presents an interesting aspect; however, it has not been directly associated with any function. It remains unknown if NM23 proteins are involved in telomere protection and therefore affect genome stability. Since telomeric damage is long known to be associated with ageing, it could be explored if NM23 proteins have a role in regulating genome stability along with the secondary conformation of telomeres, in association with TRF1/2 [83]. Moreover, do NM23 proteins also interact with other proteins of the shelterin complex? The shelterin is also known to change composition based on physiological conditions [84]. Do NM23 proteins have any role to play in this aspect? Further, it would be interesting to study if NM23-H2, in association with TRF2 or independently, binds throughout telomeres, or not. 

The interaction of NM23 proteins with telomere binding proteins such as those involved in telomere stability (TRF2) [85] and maintenance (telomerase) [86] has not been associated with the enzymatic activity of NM23 proteins. It might be possible that the kinase activity of NM23 proteins impacts TRF2/telomerase function, as both TRF2 and telomerase are known to be phosphorylated, and have phosphorylation-dependent functions [87]. 

Kar et al. demonstrated the nuclear interaction of NM23-H2 with hTERT that was found to be independent of DNA binding [5]. However, hTERT is also known to localize in mitochondria and activate mitophagy [88]. While the mitochondrial localization of NM23-H2 is not known, NM23-H4 is a mitochondrial NDPK [89]. It might be interesting to study whether NM23-H4 associates with hTERT in mitochondria, and if this association has any role in mitochondrial functions. 

hTERT is also known to activate the transcription of several gene targets [90,91]. The interaction of NM23 with hTERT, therefore, may influence transcriptomic alterations. Together, this raises the question about whether, and if so, how, the nucleic acid-independent interactions of NM23-H2 influence gene expression across the genome.

Another important aspect of NM23-H2 is its association with TRF1/TRF2. These interactions might be crucial in genome-wide telomere length-dependent transcriptome changes. This is because, recently, the extra-telomeric association of TRF2 was shown to extensively impact genome-wide gene expression changes that were dependent on telomere length [92].

Additionally, similar to NM23-H2, TRF2 is also shown to bind to G-quadruplexes and gene promoters carrying potential G-quadruplex forming sequences [93,94,95]. Therefore, NM23-H2-TRF2 interaction suggests potential co-regulation through association with G-quadruplexes. An example of this is the regulation of hTERT, where independently both NM23-H2 [12] and TRF2 [96] have been observed to control hTERT by binding to promoter G-quadruplexes. 

We further highlight the indications from the association of NM23-H2 with lamins and FEN1 [12]. TRF2 is also known to interact with A-lamins, thereby mediating the chromosome looping of telomeres [97,98]. In this light, further understanding of Lamins/TRF1/2/NM23-H2 or FEN1/NM23-H2 associations might provide meaningful insights into the role of NM23 proteins in telomere biology. 

Finally, multiple pieces of evidence of the mitochondrial NM23 protein suggests an association with ageing. Simultaneously, observations on the cytoskeleton, NM23 family proteins, telomeres, and ageing are connected. These suggest potential roles of NM23 proteins that remain to be addressed. Further work will be required to illuminate these molecular interactions and the implications of such cross-talk, if any.

## 6. Conclusions

NM23 proteins have multiple activities and functions that assist their metastasis suppressor role. While some of these are well understood, others continue to be explored. Here, we discussed the interactions and regulatory effects of NM23 proteins related to telomeres and telomere-binding proteins (Table 1). The emerging literature on NM23-H2 and NM23-H5-like proteins with telomeres suggests the possibility of telomere binding by other NM23 proteins. 

The dual regulation of telomerase, both at the transcriptional and post-translational levels, by NM23-H2 emphasizes its regulatory effect on telomeres and telomere-associated functions, including metastasis and ageing. While based on current findings it is difficult to say whether NM23 proteins have any role in these aspects, it would be interesting to investigate the reasons and implications of NM23 association with telomeres and telomere binding proteins.

Additionally, the role of NM23 proteins needs to be further explored with reference to ageing-associated pathologies. From the association of NM23 proteins with the initiation/progression of diseases such as Alzheimer’s, progeria and cancer, it is possible that NM23 family members might influence pathological contexts. Direct exploration of these questions would be important.

## Figures and Tables

**Table 1:** Summary of telomere-associated and ageing-related observations of NM23/NDPK proteins.

NM23 Protein	Telomere Binding	Interaction with Telomere Associated Protein	Implication in Ageing and Cancer
NM23-H1	✕ [6]	✕ [6]	√ [48,49,50,61,62]
NM23-H2	√ [5,6]	√ [5,12,30,31,32]	√ [12,61]
NM23-H4	-	-	[46,47]
NM23-H2 homolog, NM23B	√ [7]	-	-
NM23-H5 homolog, NME5-like Cc	√ [8]	-	-

✕: Studies report lack of interaction; √: Studies report interaction; -: No studies done in this respect.
